# The Impact of Human Mobility on Regional and Global Efforts to Control HIV Transmission

**DOI:** 10.3390/v12010067

**Published:** 2020-01-06

**Authors:** Emily A. Eshraghian, Sepideh N. Ferdos, Sanjay R. Mehta

**Affiliations:** 1 Department of Family Medicine and Public Health, University of California San Diego, La Jolla, CA 92093, USA; eeshragh@ucsd.edu (E.A.E.); sferdos@ucsd.edu (S.N.F.); 2 Departments of Medicine and Pathology, University of California San Diego, La Jolla, CA 92161, USA; 3 Department of Medicine, San Diego Veterans Affairs Medical Center, San Diego, CA 92161, USA

**Keywords:** HIV, transmission networks, molecular epidemiology, prevention and care, disease surveillance, public health response

## Abstract

HIV prevention and control methods are implemented on different scales to reduce the spread of the virus amongst populations. However, despite such efforts, HIV continues to persist in populations with a global incidence rate of 1.8 million in 2017 alone. The introduction of new infections into susceptible regional populations promotes the spread of HIV, indicating a crucial need to study the impact of migration and mobility on regional and global efforts to prevent HIV transmission. Here we reviewed studies that assess the impact of human mobility on HIV transmission and spread. We found an important role for both travel and migration in driving the spread of HIV across regional and national borders. Combined, our results indicate that even in the presence of control and preventive efforts, if migration and travel are occurring, public health efforts will need to remain persistent to ensure that new infections do not grow into outbreaks.

## 1. Introduction

Globalization and international migration are important factors that facilitate the spread of infectious diseases [1,2,3]. Human mobility promotes the movement of diseases and the establishment of epidemics in susceptible populations, leading to outbreaks of emerging and reemerging infectious diseases [1,4]. More specifically, human mobility has been identified as a key driver in the diffusion of the HIV epidemic within nations [5,6] and around the world [7,8]. Surveillance and understanding of human migration trends is important when monitoring the epidemiology of HIV in order to better understand exposure, which modifies infection rates among populations [1,9].

Communities around the globe have varying levels of prevention and control resources for reducing the transmission of HIV. Risk for acquisition and transmission is impacted by social, structural, and population level factors [7,10]. These elements contribute to local risk contexts that lead to variations in the global spread of the virus. Such risk contexts can also be impacted by the presence or absence of appropriate prevention tools and services throughout communities [7], and may be further influenced by human mobility [11].

The HIV epidemiological curve is shaped by an increasingly mobile global population, and such mobility may exacerbate the risk of HIV transmission [12]. To prevent dissemination of HIV, some countries restrict entry of HIV positive travelers [13]. However, in this era where HIV has become an easily treatable chronic disease and in which there is increasing global interconnectedness, these approaches impinge on human rights. Still, without understanding the role of mobility and migration in local HIV epidemics, the effectiveness of prevention and control efforts designed to lower HIV incidence are limited [14]. In this perspective paper, we highlight the impact of human migration and mobility on existing prevention efforts in the context of HIV dissemination.

## 2. Methods

To allow the incorporation of relevant literature as evidence for this perspective paper, searches of PubMed and Google Scholar were conducted in May 2019. The following limits were applied: Published online and in the English language between 1998 and 2019. Appropriate synonyms and free terms were used in each database search. A combination of the following search terms were used: Communicable disease, and/or migration, and/or HIV, and/or AIDS, and/or transmission, and/or transmission network, and/or importation, and/or exportation, and/or spatial clustering, and/or prevention, and/or molecular epidemiology, and/or viral diseases, and/or border surveillance, and/or spatial clustering, and/or transmission route(s), and/or travel, and/or surveillance, and/or control and/or epidemic, and/or outbreak, and/or viral migration. Further, only empirical and observational studies are included.

After database searches, we complied a total of 390 studies. Eighty-four duplicates were removed, leaving us with 306 studies to screen. Since our goal was to evaluate the impact of migration and mobility on HIV epidemiology, an additional 323 studies were eliminated upon review of titles and abstracts. Upon assessment of the full-text of the remaining 37 articles, 16 articles were eliminated based on lack of access to full-text or irrelevance to our main aims. The final 21 studies included as evidence in our paper are summarized in Table 1.

## 3. The Role of Cross-Border Coordination in Infection Control 

Infectious diseases and particularly viral pathogens have long been associated with spread due to human mobility, even in the presence of effective preventive and control efforts. In the following section, we outline pertinent examples of viral transmission impacted by mobility. The measles outbreak in 2019 demanded the attention of the entire world. Although a vaccine preventable disease, measles incidence grew rapidly across the globe in 2019 [15]. A review of measles in developed countries, including the Americas, Europe, and Australia found that 100% of measles cases were imported or import-related [16], Interestingly, in another study, the risk of importing measles was higher among indigenous travelers compared to immigrants [17], suggesting that mobility (i.e., travel) and not just migration is important in the spread of disease. 

Similarly, the Ebola outbreak in west Africa in 2014–2016 spread rapidly regionally with movement of individuals between rural and urban areas in Sierra Leone, Guinea, and Liberia [14]. This led the international community to restrict air travel from the area, which modeling studies showed was vital for preventing dissemination of Ebola to the rest of the world [18]. However, even with this, between November 10, 2014, and July 12, 2015, the US Centers for Disease Control and Prevention (CDC) made 2374 notifications of travelers who were monitored by US health departments because they had been to an Ebola-affected country in West Africa [19].

In contrast to measles and Ebola, transmitted via airborne and contact routes respectively, HIV is transmitted through contact with infected body fluids. Therefore, risk factors for HIV transmission are predominantly of a behavioral nature. While HIV is not as directly impacted by mobility as measles and Ebola, mobility and migration is often linked with behavioral mechanisms that include increased risk taking, which can impact HIV transmission [20]. For example, participation in injection drug-use [21], transactional sex [20], and rape [22] are more common among mobile populations, all of which are risks for HIV transmission. Furthermore, the latency period before clinical symptoms also allows a greater window of travel while infectious. Human migration from the displacement and movement of large refugee populations arising from areas of conflict, war, or economic hardship, also can increase risk of HIV transmission and acquisition [5,11,12,20]. Globalization itself has also historically been shown to play a crucial role in the spread and rate of infectious diseases among different populations [23,24]. In the United States, efforts focused on prevention of disease transmission and dissemination have produced substantial successes. Incident cases of HIV have been reduced through advancements in HIV testing technologies and implementation of evidence-based behavioral change interventions, surveillance of blood supply, condom use, needle exchange programs, and pre-exposure prophylaxis, among other preventive interventions [25]. Despite efforts towards control, prevention, and treatment methods for HIV through the implementation of international mechanisms and funds directed towards lower-income countries, HIV incidence continues to be unacceptably high [26].

## 4. Human Mobility and HIV

Historical lessons of responses to the HIV epidemic have illustrated that the following conditions are essential for effective control and prevention: Coordinated national planning efforts, implementation of evidence-based strategies that correspond to the population’s needs, and an environment conducive to the social and political inclusion of people living with and at risk of HIV [27]. In order to implement effective infection control and prevention efforts, it is vital to understand the dynamics of HIV transmission across regional and international borders and how these epidemics are linked [28]. To demonstrate the need of coordinating public health efforts internationally, we have formulated a volume of literature that indicates the value of and suggests the urgency of intra and international coordination efforts across borders in order to optimize infection control. Table 1 characterizes a collection of studies that place a particular emphasis on enjoining efforts across borders to recognize groups of individuals who are at an increased risk of contracting the disease. We use this literature to highlight that HIV prevention measures on one side of a border may provide only limited benefit if they are not matched across borders. It is important to note that the lack of standards in inferring linked HIV infections make it difficult to compare and contrast HIV transmission clusters across studies.

The articles included in this review discuss the impact of international migration and travel on trends in HIV epidemiology and provide key insights on this relationship. Studies looking at both regional and global transmission are included to demonstrate how HIV moves through populations on different geographic levels.

Several articles discuss the public health consequences of viral importation, exportation, or both. Many of these articles highlight the plausibility of viral importation in triggering HIV epidemics. A phylodynamic study by Rasmussen et al. found that viral introductions into KwaZulu-Natal, a rural region in South Africa, were a major driver of the ongoing epidemic, accounting for an estimated 35% of new viral infections in the region [40]. These study highlights the role of regional mobility between communities in sustaining HIV epidemics. Expanding to international travel, a study conducted in France assessed the frequency of travel-related illnesses among residents of France who had returned from abroad with an infectious disease [33]. This paper found that of the 265 cases of infections contracted by travelers, six were incident cases of HIV [33]. While this study did not examine transmission after returning to France, there is evidence that travelers can disseminate HIV once they return home [38,40]. Such evidence suggests that efforts to control HIV transmission in a region or nation need to account for viral introductions into the region of interest. Knowledge of HIV import will allow jurisdictions to better comprehend the success of their prevention and control methods [49,50] as well as maintaining vigilance to prevent emergence of new outbreaks in areas where HIV is already endemic. Healthcare providers should also consider the possibility of HIV acquisition when evaluating returning international travelers, particularly as people are known to take greater risks when traveling [33].

Human migration also has a key influence on HIV transmission. In a study of HIV across Mesoamerica, human mobility in part due to economic migration likely bridged infected and susceptible populations resulting in transmission of HIV [31]. Wertheim’s global look at the overall HIV transmission network found many links between persons from different nations, highlighting the role of travel or migration in the spread of the virus [48]. Several studies have also highlighted the onward transmission of non-B HIV-1 subtypes among migrants [45,51]. As non-B HIV-1 is much more likely to have been acquired outside of the US and other western nations, these likely represent easily identified viral introductions crossing international borders [45,51]. Additional evidence for this comes from observations of new viral subtypes [30] and circulating recombinant forms entering certain geographic regions [29]. Others have reported on how migration of infected persons into highly susceptible populations (e.g. injections drug users) can fuel new epidemics [5,36]. It is clear that human mobility and migration are important factors that must be accounted for in order to lower the burden of HIV infection [42] and allow optimization of prevention efforts. Such pathways of viral dissemination warrant intervention strategies that address mobile populations.

Since the virus can be transmitted by a person with HIV (PWH) when traveling, or acquired by a traveler who is engaging in risky behavior, it is evident that dissemination and transmission of HIV is bidirectional [44], indicating that the virus does not discriminate based on direction of movement. Considering the high rate of human mobility at border regions, this would suggest that border populations should be classified as at-risk populations, even if only side of the border has what the WHO considers a generalized epidemic (≥1.0% HIV prevalence) [50]. Allocation of resources and prevention efforts directed to border populations and across borders, may have a greater impact than the disbursement of such resources among non-mobile populations. Furthermore, with the constant influx and outflux of new infections into a regional epidemic, there is a need for consistent vigilance of these patterns to keep incidence rates low on all sides of a border. Epidemiological studies have illustrated the possibility of high frequency of extra-community HIV introductions, and suggest that that repeated introductions of HIV play a significant role in sustaining HIV incidence [52]. Some experts suggest that regulation of cross-border travel is necessary to halt the introduction of diseases and infections into regions and ensure minimal risk of an epidemic occurring or expanding [53]. However, such approaches would be nearly impossible to enact in today’s age of globalization. Evidence suggests that infection control is optimized when efforts are coordinated across borders [36,46,47], both regional and international. 

Without first understanding the dynamics that sustain transmission of HIV, prevention and control efforts can only be limited in effectiveness [10,44]. To suppress the HIV epidemic, steep reductions in incidence are critical [14]. Major reductions in incidence have been demonstrated as a result of universal antiretroviral therapy [49]. However, randomized trials demonstrate that implementing such efforts at population levels has shown insignificant differences in HIV incidence [54,55,56]. The impact of human mobility on such trials is not well understood and may pose a risk for lowering the measured effectiveness of control efforts. To some degree, migration may be acting as an effect modifier. Studies of prevention efforts that do not acknowledge and measure the role of human mobility in HIV and viral migration on outcomes are unlikely to provide accurate results of their impacts. In fact, the influence of mobility and migration may be so significant that the impact of the intervention may be lost [14]. A recent trial found that universal testing in tandem with antiretroviral treatment had the potential to lower HIV incidence, but the authors also recognized that the benefits may be compromised if HIV transmission is concentrated in subgroups [14]. Phylogenetic studies that assess the impact of mobile populations are needed to provide a more explicit understanding of the impact of viral migration on HIV prevention and control efforts.

## 5. Conclusions

The collection of studies presented in this review support coordination of prevention efforts across regional and international borders. While optimal prevention interventions for HIV transmission need to account for behaviors and barriers to care, the goal of population-level reduction and eradication of HIV will only be achieved if the impact of human mobility is properly accounted for in these settings [57]. While the classification of internationally mobile populations as high-risk groups may be useful to identifying HIV earlier in these populations, it is important to be aware that mobility occurs regionally as well, and as such care needs to be taken not to stigmatize these populations. Understanding the importance of mobility in a regional epidemic will allow regional HIV prevention programs to better estimate and plan the impact of locally deployed interventions, and help legislators understand the importance of cross-regional coordinated efforts. These need to be balanced with the rights and concerns of migrant populations. Finally, this review highlights that even in the presence of control and preventive efforts, if migration and travel are occurring, public health efforts will need to remain persistent to ensure that new infections do not grow into outbreaks. As Barnett and Whiteside have illustrated in their book *AIDS in the Twenty-First Century: Disease and Globalization*, “health and wellbeing are international concerns and global goods, and inherent in the epidemic are lessons to be learned regarding collective responsibility for universal human health” [24].

## Figures and Tables

**Table 1:** Summary of articles reviewed regarding human mobility and HIV.

Author (Year)	Relevant Aims	Results	Comments/Notes on Migration
Aibekova (2018) [29]	Analyze the distribution of HIV-1 subtype A in thirteen former Soviet Union countries	HIV-1 subtype A clusters are intermixed	Intermixed clusters indicate a possible role of migration-associated HIV transmission
Castley (2017) [30]	Determine HIV-1 subtype distribution and phylogenetic structure in Australia between 2005 and 2012	HIV-1 epidemic in Australia is characterized by an increasing prevalence of non-B subtype infections and an overall expanding subtype diversity	Migration and overseas travel are potentially associated with the increasing prevalence and subtype diversity of infections
Chaillon (2017) [31]	Understand regional HIV epidemics, the viral transmission links between these epidemics, and risk groups across the Mesoamerican region	Infrequency of international clusters suggests moderate migration between HIV epidemics of various Mesoamerican countries, but analyses indicate that Central and Southern Mexico and Belize were significant sources of HIV transmission throughout Mesoamerica Evidence of significant viral migration within Mexico	Human migration or travel is assumed to be the cause of transmission links among individuals from different nations Such transmission links may impact the overall epidemic by bridging susceptible populations and resulting in onward transmission
de Pina-Araujo (2015) [32]	Reconstruct the phylogenetic relationship, onset date, and dissemination routes of the HIV-1 subtype G clades present throughout Angola, Cape Verde, and Portugal	HIV-1 subtype G likely originated in Central Africa and dissemination to Western and West-Central Africa occurred about a decade later	The local dissemination of HIV-1 subtype G infections in Cape Verde and Portugal are shaped by historical and ongoing movement of human populations between Angola, Cape Verde, and Portugal
Leroy (2008) [33]	To assess the frequency of travel-related illnesses among ill-returned travelers in one infectious diseases department in France	Of the 265 diagnoses made in travelers who returned with illness, there were 6 incident HIV cases	Travelers are susceptible to infections, which have the potential to be disseminated when they return to their home country
Magiorkinis (2016) [34]	Clarify the global routes of the HIV epidemic & understand the influence of human activity on HIV dissemination	The American continent and the Caribbean drove the epidemic outward to other geographic locations through an initial random migration event and further subsequent migrations globally	Dissemination of HIV through specific migration routes are consistent with geopolitical factors that affected human activities during the last 50 years, including migration. This evidence supports the argument that epidemic control policies should be implemented at global scales
Mehta (2015) [28]	Characterize HIV transmission networks in the San Diego–Tijuana region using HIV sequence and demographic data	Persistent bidirectional cross-border HIV transmission link groups across the border	Viral migrations of HIV across the border stemmed from recently formed transmission clusters, which may be indicative of ongoing cross-border transmission The HIV epidemic is relevant and important to at-risk communities on both sides of a border
Paraskevis (2009) [35]	Infer the migration history of HIV-1 subtype B among 17 countries in Europe	In most countries, the epidemic was introduced by multiple sources and subsequently spread within local networks; considerable differences between countries are present	Considering the pathways of viral spread together with the geographic distributions of viral phylogenies, intervention strategies should also address mobile populations
Paraskevis (2017) [36]	Estimate the proportion of postmigration HIV-1 transmissions among migrants who inject drugs & infer whether postmigration transmission occurs more frequently through contacts with other migrants who inject drugs or through contacts with Greek injection drug users	A majority of HIV-1 infections in migrant injection drug users were acquired after migrants moved to Greece Transmission of HIV-1 occurs more frequently through contacts between migrants	Migrant populations are a priority for targeting HIV prevention efforts, as there exists significant transmission networking among migrants subsequent to their migration
Paraskevis (2019) [37]	Identify and characterize molecular transmission clusters using molecular phylogenetic analyses & examine impact of clinical and demographic factors on regional phylogenetic clustering	Local populations are more likely to be infected within their own country, as reflected by the finding that subtype B infections have a higher probability of belonging to a molecular transmission cluster	Growth of regional HIV-1 epidemics is mainly associated with recent molecular transmission clusters compared to established cases
Parczewski (2014) [38]	Investigate the HIV sequence variability among travel-associated cases with reported infection outside of Poland and trace the country of origin where infected was transmitted	Import of HIV-1 non-B subtype variants, including recombinant viral forms, is frequent among travelers	Introduction of HIV into the local population by immigrating travelers may contribute to an increasing number of circulating variants and viral diversification, while posing a risk of exponential infection spread in the population
Pieniazek (1998) [39]	Determine the spread of HIV-subtype pattern in Lebanon, where the disease is not endemic but slowly increasing and where there is frequent population movement to and from other countries	Phylogenetic clusters used to track molecular and epidemiologic data indicate multiple HIV subtype introductions within Lebanon, and indicate that most male patients were infected abroad with the HIV-1 strains most common in the geographic location that they traveled to	Results show genetic variations in HIV within Lebanon and the discovery of new HIV-subtypes which indicate extreme heterogeneity of HIV subtypes in Lebanon
Rasmussen (2018) [40]	Quantify the contribution of local viral transmission versus external introductions to overall HIV incidence	The majority of samples are interspersed throughout clades composed predominantly of external samples, indicating that many independent introduction events have occurred into the local population from elsewhere	External viral introductions play a large role in sustaining high HIV incidence, which confirms the important role of human mobility in HIV dissemination
Schlagenhauf (2015) [41]	Assess available European surveillance data for travel-related illnesses to profile imported infections, track trends, identify risk groups, and assess the usefulness of pre-travel advice	Migration within Europe was associated with HIV/AIDS infections, among several other infections Pre-travel consultation was associated with significantly lower proportionate morbidity ratios for HIV/AIDS	The profile of travel-related infections varies according to the traveler type, migration trends, and the purpose of travel Pre-travel advice is useful for reduction of proportional morbidity with several infections, including HIV/AIDS
Sinka (2003) [42]	Describe the epidemiology of HIV infection acquired in Africa and among African communities in the United Kingdom	African-acquired HIV infections diagnosed in the United Kingdom by 2001 make up about 21% of all reported infections and continue to increase rapidly specific to different regions within Africa	Early diagnosis of HIV infection is an important component of intervention to prevent transmission, but migration, diagnosis of long-standing infection, and incident cases are potential influences that should be accounted for in order to lower the burden of HIV infection
Skar (2011) [43]	Investigate the dynamics of HIV-1 transmission among injection drug users during the outbreak in Stockholm & whether the outbreak could be due to the introduction of a new, more transmissible HIV-1 variant	The HIV outbreak that took place in Stockholm resulted from an imported variant	The outbreak was likely caused by the introduction of HIV into a network of injection drug users who were previously uninfected, which poses a valuable avenue for preventive efforts
Takebe (2014) [44]	Explore possible linkages between the HIV epidemics in East Asia and those in the rest of the world	HIV subtype B variant that was common among Japanese MSM was detected worldwide, suggesting that specific subgroups may have been circulating locally before disseminating to other countries through global contact networks	Viral migration is bidirectional, as seen by trends in countries whose HIV epidemics have been considered a result of imported viruses, but have demonstrated exportation of specific HIV lineages
von Wyl (2011) [45]	Examine the possibility of locating evidence for ongoing non-B transmission in Switzerland by identifying possible transmission pairs	Migration has a greater contribution to newly diagnosed non-B subtype HIV-1 infections among Africans compared to domestic transmission	Migration and domestic transmission both play a role in the epidemiology and transmission of non-B subtype HIV-1 A fraction of all non-B infections diagnosed in Switzerland could be prevented by local interventions To contain HIV dissemination, awareness should be raised among immigrants and Swiss individuals with partners from countries where HIV is endemic
Wang, X (2015) [46]	Determine efficiency of using inferred network characteristics to target prevention interventions	The efficiency of targeting prevention efforts according to the number of network connections was higher than if not targeting such efforts	Using network connectivity information to administer preventive treatment may be an efficient way to deliver prevention interventions and will aid in lowering rates of HIV dissemination
Wang, Y (2015) [47]	Assess HIV-1 prevalence and subtype distribution of entry travelers at the HeKou port, a major land port between Yunnan and Vietnam, from 2003 to 2012	Trends of the HIV-1 molecular epidemiology among cross-border travelers within the HeKou port highlights the importance of continually monitoring new trends of the HIV epidemic and emerging novel recombinants, particularly among migrating populations	To prevent transmission of infectious pathogens, extended epidemiologic surveillance should be implemented at borders, especially where cross-border populations have particularly high mobility
Wertheim (2014) [48]	Analyze global HIV-1 transmission patterns through construction of HIV-1 transmission clusters using only close genetic links to identify potential transmission partners	Individuals from 68% of included countries/regions had potential transmission partner(s) from another country/region	The extent of international linkage among HIV subtypes suggests the need to consider the global diversity in HIV when describing epidemics
